# The role of antimullerian hormone in assessing ovarian damage from chemotherapy, radiotherapy and surgery

**DOI:** 10.1097/MED.0000000000000447

**Published:** 2018-10-18

**Authors:** Queenie Ho Yan Wong, Richard A. Anderson

**Affiliations:** a Department of Obstetrics and Gynaecology, Queen Mary Hospital, The University of Hong Kong, Hong Kong; b Department of Obstetrics and Gynaecology, Princess Margaret Hospital, Hong Kong; c MRC Centre for Reproductive Health, Queen's Medical Research Institute, University of Edinburgh, Edinburgh, UK

**Keywords:** antimullerian hormone, chemotherapy, endometriosis, ovarian surgery, radiotherapy

## Abstract

**Purpose of review:**

Iatrogenic ovarian damage can occur after chemotherapy, radiotherapy and surgery for cancer as well as for non-malignant conditions. This review describes the effects of such treatment on antimullerian hormone (AMH) and the implications of the fall in AMH in relation to ovarian function and fertility, especially in the era of improved fertility preservation strategies.

**Recent findings:**

The risk of gonadotoxicity differs between chemotherapy regimens. There is growing evidence that pretreatment AMH has prognostic significance for the degree of fall in AMH after treatment, the reversibility of ovarian damage and risk of premature ovarian insufficiency. The accuracy of prediction increases when age is coupled with AMH. The adverse effect of removal of endometriomas is increasingly clear, and AMH pre and post surgery useful is assessing the degree of damage to the ovary. The implications of low AMH after such treatment on natural fertility and reproductive lifespan are less clear. Apart from treatment effects, there are other coexisting conditions that can affect AMH which needs to be taken into consideration during interpretation of AMH before and after treatment.

**Summary:**

A fall in AMH in women after gonadotoxic treatment has been consistently described, with variable recovery, the accurate interpretation and clinical application of post-treatment AMH level on reproductive lifespan and fertility prediction needs to be studied in future larger prospective studies with longer follow-up.

## INTRODUCTION

Iatrogenic ovarian damage can occur with chemotherapy, radiotherapy and surgery for cancer, for non-malignant conditions such as conditioning for stem cell transplant in haematological diseases, and in gynaecological conditions such as endometriosis. Depending on age at treatment, impairment of ovarian function because of accelerated depletion of primordial follicles has implications for puberty, fertility and the long-term health consequences of oestrogen deficiency as it may result in a woman living more than half her life in a postmenopausal state. It is important for clinicians to be able to detect and perhaps predict ovarian impairment early to optimize the management of these girls and women, allowing fertility preservation to be offered to those women who are at significant risk of permanent ovarian damage before they undergo gonadotoxic treatment such as total body irradiation (TBI) or high risk chemotherapy [1]. This review focuses on the use of antimullerian hormone (AMH) in assessing the effects on the ovarian reserve of these gonadotoxic treatments. 

**Box 1:**
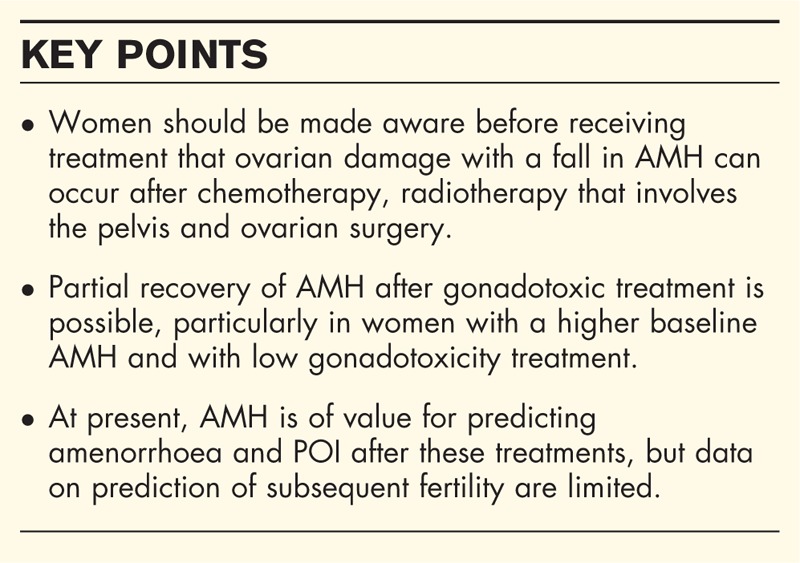
no caption available

### What is the ovarian reserve?

The term ovarian reserve refers to the population of primordial (nongrowing) follicles in the ovary. It is also used as a convenient short-hand in reproductive medicine to mean the number of follicles that can be stimulated by exogenous ‘functional’ ovarian reserve, and to describe the reproductive potential of a woman as a function of the number and quality of her oocytes [2], but these usages blur the important distinction between the true ovarian reserve and the size of the pool of growing, generally small antral, follicles. Although many chemotherapy regimens result in loss of growing follicles, it is the effect on the nongrowing pool that is important for a woman's future reproductive function. The number of primordial follicles peaks in foetal life at 18–22 weeks with 300 000 primordial follicles per ovary. By birth, the number of primordial follicles in the ovaries has already started to fall: this continues through pre and postpubertal life with only 12 and 3% of nongrowing follicles left at 30 and 40 years of age, respectively. At menopause, only around 1000 follicles remain [3,4].

### Ovarian reserve testing with serum antimullerian hormone

AMH is considered the best currently available biomarker of the ovarian reserve, although it only reflects this indirectly [5]. It is a glycoprotein which is produced by secondary, preantral and early antral follicles up to 9 mm diameter. Although AMH is not produced by primordial follicles, its circulating level is significantly correlated with them in healthy women [6]: importantly, this is based on only a small number of samples. There is an initial rise in AMH shortly after birth, then a gradual continuous increase throughout childhood with some fluctuation around puberty with a peak at 25 years [7,8]. That AMH is readily detectable in prepubertal girls makes it a potential useful marker [9], though its interpretation in childhood and adolescence is more challenging because of the physiological rise in AMH until age 25 [7]. Thereafter, AMH decreases with age, and initial studies suggested that it becomes undetectable around 5 years before menopause [10]. Thus, an undetectable AMH indicates transition to menopause. With the emergence of more sensitive AMH assays, the interval between undetectable AMH and last menstrual period will be reduced but this has yet to be defined clearly.

AMH has little inter-cyclical [11] and intra-cyclical variation as it reflects the number of 5–8 mm diameter rather than preovulatory follicles [12] thus conveniently can be assessed throughout the menstrual cycle. The most established clinical use of AMH is prediction of ovarian response to ovarian stimulation in assisted reproductive technology, predicting oocyte quantity but not live birth [13].

### Effects of chemotherapy on ovarian function and antimullerian hormone

The potential use of AMH to detect a detrimental effect of chemotherapy on ovarian function was first described in a group of childhood cancer survivors [14] who had lower AMH levels despite regular menstrual cycles, compared with healthy women. The effects of chemotherapy on AMH in women with cancer were subsequently demonstrated during treatment for breast cancer with a rapid and drastic fall during chemotherapy and limited recovery thereafter [15]. This has since been confirmed [16–19], with AMH often becoming undetectable after the first cycle of chemotherapy. By the end of six cycles of chemotherapy treatment for breast cancer, AMH is almost undetectable in most women [17].

**FIGURE 1:**
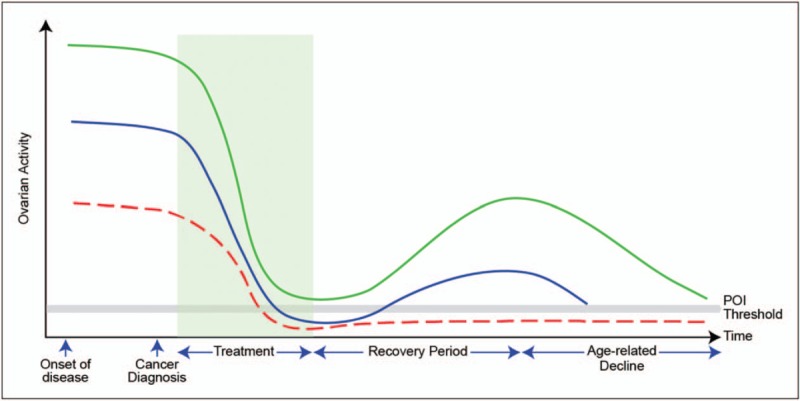
Schematic of the effect of gonadotoxic cancer treatment on ovarian function. The three lines represent women with high, average and low ovarian reserve (as, e.g. reflected in AMH concentrations). Treatment results in a rapid fall in all women. Those with low ovarian reserve (red line) are more likely to develop POI during treatment, and for that to persist thereafter. Conversely, those with higher ovarian reserve will show a variable recovery, some going on to develop early POI (blue line) with others, at the highest level of ovarian reserve (green line) showing more prolonged ovarian activity, with later the normal age-related decline. POI, premature ovarian insufficiency. Reproduced with permission [26].

**FIGURE 2:**
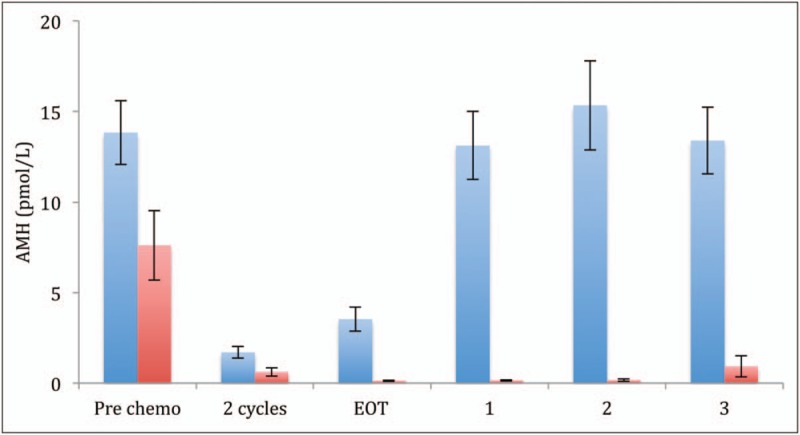
AMH concentrations at prechemotherapy, after two cycles of Doxorubicin, (Bleomycin), Vinblastine, Dacarbazine [A(B)VD], at end of treatment and at 1, 2 and 3 years after chemotherapy. Blue, women treated with A(B)VD throughout; red, women treated with BEACOPP after 2 cycles of A(B)VD. Mean ± sem. A(B)VD, Doxorubicin, (Bleomycin), Vinblastine, Dacarbazine; AMH, antimullerian hormone. Adapted from [28].

The degree of ovarian damage depends on the type of chemotherapeutic agents used, cumulative dosage and age at the time of treatment. Alkylating agents are associated with the highest risk of gonadotoxicity in terms of higher risk of low AMH, amenorrhoea and lower chance of recovery of AMH [20,21]. AMH is undetectable and shows no recovery in women receiving high-risk treatment: women and children treated with alkylating agents in high doses showed only little recovery of AMH [9,16]. Age has an important impact, with younger women have a higher pretreatment AMH, and a lower risk of undetectable AMH at the end of treatment chemotherapy (65% in women treated for breast cancer aged over 40; 16% in women aged ≤40 years) [22^▪▪^]. Younger age and higher pretreatment AMH brings a lower risk of chemotherapy-related amenorrhoea [17,19,22^▪▪^,^23^] and higher chance of return of menstruation [24,25]. AMH recovery is slower in older aged women [18,22^▪▪^]. The rate of recovery of AMH is faster if basal AMH is higher [16]. Pretreatment AMH therefore predicts post-treatment recovery of ovarian function and AMH levels, which in turn indicate remaining ovarian lifespan. Figure 1 illustrates the effect of gonadotoxic treatment on ovarian activity as reflected in AMH levels over time [26]. Women treated with ‘low risk’ chemotherapy also show a fall in AMH to low levels during treatment [27,28] but with good recovery thereafter (Fig. 2). A recent analysis has however demonstrated that recovery is limited in women aged more than 35 years, with that loss of recovery related to age and not pretreatment AMH [28].

### Effects of radiotherapy on ovarian function and antimullerian hormone

Oocytes are very sensitive to radiotherapy [29]. The detrimental effects depend on irradiation field, dosage, fractionation schedule and whether the patient is pre or post menarche. There are more limited data on AMH after radiotherapy than after chemotherapy. Childhood cancer survivors who received radiotherapy to the abdomen, pelvis, sacrum and total body have lower AMH than survivors with irradiation to other parts of the body (<0.1 μg/l versus 1.5 μg/l) [30]. Women treated with TBI for stem cell transplant all developed premature ovarian insufficiency (POI) with undetectable AMH [31], and TBI as part of the conditioning therapy before bone marrow transplant affects AMH more than chemotherapy or radiotherapy for childhood malignant or non-malignant disease [32].

### Effects of surgery on ovarian function and antimullerian hormone

Ovarian cysts are common in reproductive age women, and laparoscopic ovarian cystectomy is a recommended treatment for benign cysts [33]. There is reduction in the ovarian reserve after ovarian cystectomy as evidenced by a fall in AMH [34,35] and partial recovery in AMH is observed in some women from 3 to 12 months after excision of endometrioma [34,36–38]. The proposed mechanisms for ovarian damage after cystectomy include inadvertent damage to or removal of underlying healthy ovarian tissue during stripping of the ovarian cyst wall [39] and two recent systematic reviews and meta-analyses suggested that the use of bipolar diathermy is associated with a greater decline of AMH when compared with nonthermal haemostatic methods including suturing or haemostatic sealant [40,41^▪^]. The risk of ovarian damage and fall in AMH after cystectomy for endometrioma is greater than after surgery for other benign ovarian cysts in some studies. However, a recent meta-analysis reported a similar magnitude of reduction in AMH (38%) after both types of cystectomy [42].

A greater fall in AMH is observed after bilateral cystectomy for endometrioma [34,35,43–45] and with surgery with damage to the ovarian blood supply [43,44,46]. There is a higher chance of disruption of the blood supply to the ovaries and therefore a greater fall in AMH when cystectomy is performed in women with a higher revised American Society of Reproductive Medicine score or when adnexal dissection involves the mesosalpinx. AMH decline is reported not to be associated with cyst size in most studies [36,43,47,48]. As with data after chemotherapy, women with a lower preoperative AMH have a higher chance of developing diminished ovarian reserve (DOR; defined as AMH <1.1 ng/ml or 7.9 pmol/l) after ovarian cystectomy for endometrioma. A preoperative AMH less than 2.1 ng/ml (15 pmol/l) or less than 3.5 ng/ml (25 pmol/l) predicted DOR (AMH <1.1 ng/ml or 7.9 pmol/l) at 6 months for unilateral and bilateral cystectomy, respectively [35].

### Prediction of amenorrhoea or premature ovarian insufficiency using antimullerian hormone

There is an increased likelihood for a healthy woman to reach menopause before the median age of 51 years if AMH is low for her age [49]. Conversely, if her AMH level is more than 0.02 ng/ml (0.14 pmol/l), there is minimal chance of menopause within 5 years regardless of women's age [50]. The application of a low AMH to predict the probability of menopause is more sensitive in late than in young reproductive age women, but an undetectable AMH level (using a high-sensitivity assay) is predictive of menopause within 5 years in only 60% of healthy women aged more than 45 years [50]. The difficulty of predicting menopause accurately by using AMH seems because of the differences in decline trajectory between different women [51], as well as other modifiers such as smoking, and at present it remains impossible to predict age at menopause accurately in healthy women.

**FIGURE 3:**
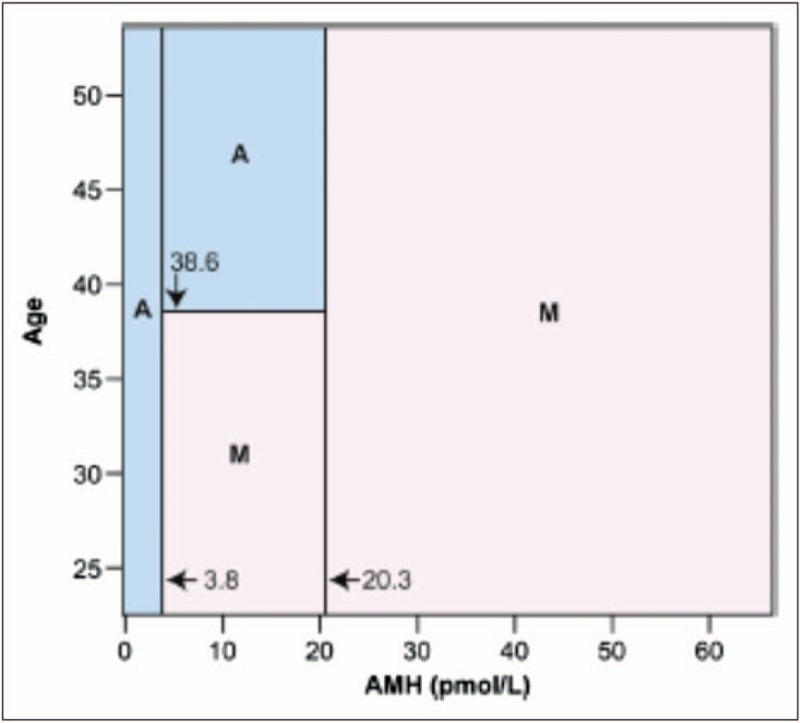
Mosaic chart for ongoing menses (M) or chemotherapy-related amenorrhoea (A) using serum AMH and chronological age as predictor variables. AMH, antimullerian hormone. Reproduced with permission [19].

The value of AMH for prediction of amenorrhoea after gonadotoxic treatment largely derives from studies in women receiving chemotherapy for breast cancer. The risk of amenorrhoea can be predicted by pretreatment AMH with the risk of persistent amenorrhoea higher with a lower pretreatment AMH. Younger age and higher pretreatment AMH are two important favourable prognostic factors for ovarian recovery [19,52,53]. Women with pretreatment AMH of less than 7.3 pmol/l were 9.3 times more likely to develop POI 2 years after chemotherapy [22^▪▪^]. A mosaic chart incorporating both age and pretreatment AMH level for postrecovery menstruation prediction indicates that, for all premenopausal women, an AMH level less than 3.8 pmol/l predicts amenorrhoea, whereas AMH more than 20.3 pmol/l predicts continuing menstruation at 2 years (Fig. 3). For intermediate values, age also becomes an important factor [19]: larger studies are needed to validate and refine this analysis. In women older than 40 years, an undetectable AMH (using a highly sensitive assay) immediately post-treatment can predict POI at 2 years with good accuracy [22^▪▪^], whereas younger women showed recovery of AMH, including from undetectable levels. In older women this may be a potential guide to post-treatment endocrine therapy, but it may not be sufficiently reliable in younger women. Whether this can be improved with further improvements to the sensitivity of AMH assays is unclear, as it will also be determined by whether there are sufficient remaining nongrowing follicles (that do not produce AMH) to support development of antral follicles that do produce AMH. There is likely to be a combination of assay sensitivity and time as chemotherapy (perhaps combined with pretreatment AMH, age and an index relating to the chemotherapy regimen) that will reliably be able to predict ovarian recovery versus permanent ovarian failure.

The possibility of natural conception in POI is 5–10% [54]. Fertility preservation should be offered to those women who are at risk of irreversible ovarian damage with no recovery of AMH before they undergo gonadotoxic treatment such as TBI and high-risk chemotherapy.

### Prediction of fertility with antimullerian hormone

Current evidence clearly demonstrates that AMH is not a ‘fertility test’, predicting neither natural conception in healthy women [55,56,57^▪▪^] nor live birth after assisted reproduction [13]. Prospective cohort studies involving healthy women predominantly in their 20s [56] or 30s [57^▪▪^] showed no difference in fecundability between women with low and normal ovarian reserve and between older and younger women with DOR.

Childhood and adult cancer survivors have reduced fertility depending on diagnosis/treatment given [58,59], and AMH in cancer survivors is also lower when compared with healthy women [14,30,60]. However, there is no direct evidence demonstrating that a lower AMH is predictive of reduced chance to conceive naturally after cancer treatment. A retrospective study reported that there was no difference in pregnancy occurrence with detectable or undetectable post-treatment AMH in 134 women treated with breast cancer [61]. Natural pregnancy has been reported even when AMH was undetectable after cancer treatment [17,62,63], although the assays used were less sensitive than current ones.

The implications of low AMH for fertility after ovarian cystectomy for benign ovarian cyst on pregnancy are similarly unclear. Women with low preoperative AMH have higher risk of DOR diagnosed 6 months after operation, and a lower cumulative spontaneous pregnancy rate at 24 months after ovarian cystectomy for endometrioma in women with DOR than non-DOR women (14.4 versus 59.2%) has been reported [35]. A prospective study found that postoperative AMH level at 6 months after ovarian cystectomy for benign ovarian cyst was not different between pregnant and non-pregnant groups [64]. It is difficult to draw any conclusion on the predictive value of low AMH after ovarian cystectomy on fertility outcome as most studies do not include pregnancy as an outcome. Interpretation of results from studies reporting pregnancy needs to be cautious as we need to know whether those women included in the studies sought to become pregnant, and the studies usually only report pregnancy as a secondary outcome.

### Other issues to consider during interpretation of antimullerian hormone

AMH GenII ELISA assay by Beckman Coulter (Brea, CA, USA) was the most commonly used assay until recently, but is increasingly being replaced by automated assays. The lower limit of detection is valuable for analysis of the very low AMH levels often found after chemotherapy. There is still a difference in calibration between assays [65] which should be taken into account if comparing studies, until an international standard to unify the calibration between different assays is available.

Table 1 shows the factors affecting AMH level that should be considered before clinical application of AMH levels in this and other contexts (reviewed more fully in other articles in this collection). Women with lymphoma have lower AMH at diagnosis than age-matched healthy women and women with other cancers before treatment [66]. In girls with a range of newly diagnosed cancers, including leukaemia, lymphoma, sarcoma, nephroblastoma and neuroblastoma, AMH level was correlated with general health markers, that is, pyrexia, C-reactive protein (CRP) and anaemia [67]. Other factors likely to be of relevance in interpreting AMH levels in this context include BRCA-1 gene carrier and the use of hormonal contraception. BRCA-1 gene mutation carriers have higher risk of developing breast cancer and their AMH is 25% lower than noncarriers [68]. Hormonal contraceptives are commonly used by this population of young women, and additionally as adjuvant treatment after surgery for endometriosis, and sometimes as hormone replacement. AMH is 19% lower in users of combined oral contraceptives than in nonusers [69]. Gonadotropin hormone releasing hormone (GnRH) agonist treatment also reduces AMH over a period of several months [15].

**Table 1:** Factors affecting antimullerian hormone level

Decreased AMH level	Increased AMH level	Inconsistent results
Older age Smoking Pregnancy History of ovarian surgery Endometriosis Cancer Use of hormonal contraception or GnRH agonist Ovarian toxic treatment (chemotherapy, radiotherapy BRCA1 gene carrier Fragile X mental retardation-1 (FMR-1) gene carrier	White ethnicity Polycystic ovary syndrome Granulosa cell tumour	High BMI Parity Vitamin D status

AMH, antimullerian hormone.

## CONCLUSION

A decrease in ovarian reserve with a fall in AMH can occur after treatments including chemotherapy, radiotherapy and ovarian surgery. In some women, depending on age, preexisting ovarian reserve/AMH and treatment administered, POI may result with undetectable AMH after treatment and without recovery. Clinicians should be aware of such effect and include discussion of the possible implications of decreased AMH on ovarian reserve and fertility with women before undergoing treatment. The accurate interpretation of post-treatment AMH level on reproductive lifespan and fertility prediction needs larger prospective studies with longer follow-up.

## Acknowledgements

We are grateful to the many colleagues who have collaborated in our studies described here.

### Financial support and sponsorship

Funding: The authors work in this field has been supported by MRC grants G1100357 and MR/L00299X/1. Part of this work was undertaken in the MRC Centre for Reproductive Health which is funded by MRC Centre grant MR/N022556/1.

R.A.A. has received research support and honoraria from Roche Diagnostics, Beckman Coulter and Ansh Labs.

### Conflicts of interest

There are no conflicts of interest.

## REFERENCES AND RECOMMENDED READING

Papers of particular interest, published within the annual period of review, have been highlighted as:

▪ of special interest
▪▪ of outstanding interest
